# Students as effective harm reductionists and needle exchange organizers

**DOI:** 10.1186/s13011-017-0099-0

**Published:** 2017-03-17

**Authors:** Kyle Barbour, Miriam McQuade, Brandon Brown

**Affiliations:** 1 UC Irvine School of Medicine, Irvine; Orange County Needle Exchange Program (OCNEP), Santa Ana, USA; 2 UC Riverside School of Medicine, Riverside, USA

**Keywords:** Needle exchange, Syringe exchange, Harm reduction, Medical students, Graduate students, Medical education, Student-run free clinics, Leadership, Advocacy, Stigma

## Abstract

**Background:**

Needle exchange programs are safe, highly effective programs for promoting health among people who inject drugs. However, they remain poorly funded, and often illegal, in many places worldwide due to fear and stigma surrounding drug use. Continued advocacy, education, and implementation of new needle exchanges are thus essential to improve public health and reduce structural inequality.

**Commentary:**

We argue that students, and especially professional and graduate students, have the potential to play an important role in advancing harm reduction. Students benefit from the respect given to the professions they are training to enter, which gives them leverage to navigate the political hurdles often faced by needle exchange organizers, especially in areas that presently lack services. In addition, due to their relative simplicity, needle exchanges do not require much of the licensing, clinical knowledge, and infrastructure associated with more traditional student programs, such as student-run free medical clinics. Students are capable of learning harm reduction cultural approaches and techniques if they remain humble, open-minded, and seek the help of the harm reduction community. Consequently, students can generate tremendous benefits to their community without performing beyond their appropriate clinical limitations.

Students benefit from organizing needle exchanges by gaining applied experience in advocacy, organization-building, and political finesse. Working in a needle exchange significantly helps erode stigma against multiple marginalized populations. Students in health-related professions additionally learn clinically-relevant knowledge that is often lacking from their formal training, such as an understanding of structural violence and inequality, root causes of substance use, client-centered approaches to health services, and interacting with clients as peers, rather than through the standard hierarchical medical interaction.

**Conclusion:**

We therefore encourage students to learn about and consider organizing needle exchanges during their training. Our experience is that students can be successful in developing sustainable programs which benefit their clients, the broader harm reduction movement, and themselves alike.

## Background

Needle exchange programs (NEPs) are the most effective public health intervention available to prevent infectious diseases among people who inject drugs (PWID) [2, 11, 22]. NEPs function by providing a safe way to dispose of used syringes, obtain clean injection supplies, and in many cases access HIV and hepatitis C testing, referrals to drug treatment, and other social services. By engaging with PWID instead of rigidly adhering to abstinence or criminalization approaches, NEPs embody a harm reduction approach and dramatically reduce infectious disease transmission rates, supported by studies showing reductions in hepatitis C by 65%, hepatitis B by 61%, and HIV by 33% among clients [16, 24, 37]. NEPs further have been shown to decrease risky behavior [6] and to provide a critical linkage to care for PWID, who may receive all or most of their services through their NEP [19]. These valuable benefits occur without any known downsides, as NEPs often reduce syringe waste found in the community [4, 9, 10, 30, 35, 39], decrease needlestick injuries [15], and have no negative impacts on crime or amount of drug use [13, 17, 26, 29, 34, 37, 38].

Despite these impressive outcomes, NEPs remain politically contentious, largely due to significant social stigma against injection drug use and fears about legitimizing drug injection or generating syringe waste. Recently, an example of this pushback made national news during a significant outbreak of HIV in Indiana, where the state government expressed significant resistance to NEPs before reluctantly lifting the statewide needle exchange ban under pressure from the public health community [31]. Although these fears have not been validated by research, they generate significant hostility, with NEPs remaining illegal in many places and forced to close due to public backlash in others [4, 5, 8, 27, 41]. Consequently, education and policy change alongside program implementation are important goals for harm reduction advocates.

Social injustices number among the most significant causes of mortality in the United States, with low education killing more people than myocardial infarction, racial segregation more than cerebrovascular disease, and poor social support more than lung cancer [7, 14, 25]. Consequently, repeated calls have been made to incorporate social justice, sociopolitical determinants of health, and understanding of structural violence into health curricula, including from the World Health Organization, American Association of Medical Colleges, American Board of Internal Medicine, American College of Physicians, European Federation of Internal Medicine, and many physicians and other health leaders [1, 3, 7, 20]. By organizing NEPs, students are able to simultaneously advance harm reduction and lead their educational institutions towards the social justice orientation recognized as being essential to promote health and equality.

Our experience as harm reductionists and NEP organizers is limited to the United States, with its educational system, laws, and culture surrounding injection drug use. In this region, NEPs remain illegal in many states and frequently opposed in others, with acceptance of harm reduction growing but far from complete. Student organization of NEPs may be easier in countries without restrictive laws against harm reduction, and much harder, although not impossible, in those with harsher regulations. Despite differences between the United States and other parts of the world, we hope that this commentary encourages all students who are concerned about health and social justice to involve themselves in the harm reduction movement to the degree possible, whether by organizing NEPs, campaigning to repeal laws limiting harm reduction, supporting other activists, or in other ways contributing to the welfare of PWID and other stigmatized groups.

## Why students should organize NEPs

We argue that students, especially students in the health professions (such as medicine, nursing, public health, and pharmacy) and related fields (such as law, sociology, criminology, social work, and many others) have the potential to play important roles in advocating for and expanding access to harm reduction services, such as NEPs. Professional students have privileges that allow, and in our view, require, that they take steps to better their community. As student projects, NEPs are often passed over – or never considered – in favor of student-run free medical clinics, where medical and other health professions students manage the clinic’s logistics and see patients (typically from underserved populations) under the oversight of a licensed clinician [32]. Many medical schools in the United States have at least one student-run free clinic, and often more. Yet as of 2016, there was only one student-run NEP in the United States – ours in Orange County, California – and only two other student-organized harm reduction initiatives, the Iowa Harm Reduction Coalition’s sterile injection supply program and the ultimately-successful push by University of Miami medical students to lift Florida’s ban on needle exchange.

However, student-run medical clinics have challenges which NEPs do not, such as needing oversight from licensed health professionals, maintenance of health records, medical equipment, a building with clinic-appropriate infrastructure and zoning, administrative staff, and other complexities. In contrast, NEPs are cheap, have little overhead, require no clinical oversight, and train aspiring health professionals in solidarity with an underserved population and on-the-ground advocacy, aspects frequently missing from clinical training, despite their importance. Consequently, student leaders have the potential to create an enormously positive impact in their community, gaining experience that will aid them throughout their careers in the process. We feel the examples mentioned above demonstrate that student-initiated efforts can be successful in a range of contexts, whether or not legal pathways to NEP approval presently exist there.

The most important aspect of any NEP is to provide empathetic, nonjudgmental harm reduction services to its clients, who often experience their NEP as one of the only safe environments in their lives [28]. Our focus here on the benefits to organizers is not to ignore that clients should be the central focus of any NEP. Rather, we recognize that students often seek opportunities to help their communities in medical and public health arenas, yet harm reduction interventions such as NEPs are often overlooked or not known to potential student leaders. Our hope here is to provide a rationale that helps students and their mentors understand how organizing an NEP is relevant to their future practices in medicine and public health. In making this argument, we hope that more students learn about harm reduction and use its lessons to improve the lives of their future patients and community.

## Knowledge and self-reliance

By definition, students are not yet able to independently manage patients. This presents a significant challenge for student-run free medical clinics, as oversight must be provided at all times, usually by faculty with little free time. In contrast, NEPs are traditionally operated by peers and require no medical training. Although students generally lack grounding in harm reduction, there are several excellent, free manuals which introduce a solid harm reduction framework [18, 40, 42, 43]. Additionally, the harm reduction culture has strong roots in activist, horizontally-organized traditions [12, 33] and is accordingly an encouraging, friendly environment to people who are humble and eager to learn. For dedicated students, reading these documents, communicating with experienced harm reductionists, and shadowing at NEPs can provide a baseline of knowledge which can grow as they build their program.

Frequently, people new to harm reduction find its core principles challenging to put into practice. Harm reduction is founded on principles of solidarity, mutual aid, and letting PWID determine their own fates; a different orientation than is frequently encountered around drug use. We encourage student organizers to embrace this learning curve and discuss issues with other harm reductionists (nearby or not) and their potential clients. Compassionately examining one’s unconscious biases and misperceptions is a crucial part of harm reduction and effective clinical practice, and doing so benefits clients, programs, and oneself alike.

As with any student-run project, setting up an NEP is not without challenges, and student NEP organizers must deal with politics, funding, space, manpower, and other potential issues. However, with the exception of politics, NEPs are substantially cheaper and simpler than clinics. This makes students capable of directly managing the programs they build, building their self-reliance, problem-solving, and leadership skills. In contrast, clinics are expensive, complex, and regulated, and consequently similar problems must often be dealt with by licensed clinicians and non-student staff. This can force students out of core decisions, alter the vision for the clinic, and cause students to lose the opportunity for self-management, an especially unfortunate cost given the few chances to work independently in most students’ training.

Students may find themselves unable to organize an NEP due to political or legal restrictions on harm reduction programs, both within and outside the United States. In these cases, students may be able to focus on political advocacy to overturn laws restricting syringe exchange, as we discuss more below, while implementing the services which are presently possible and building the infrastructure which could support an NEP in the future. These services include HIV and hepatitis C testing, naloxone distribution and overdose prevention education, linkage to other resources, and distributing sterile injection supplies other than needles (such as cottons, cookers, sterile water, and others). These services are similarly logistically simple and have many of the benefits of NEPs to clients and organizers alike, and can be a crucial stepping stone in regions where NEPs remain illegal.

## Political leadership

Compared to clinics, the one area where NEPs are generally more complex is politics. However, students are capable of navigating significant political hurdles, and have successfully campaigned to overturn laws banning harm reduction programs [36] or open NEPs in regions hostile to harm reduction, such as in our case. In doing so, students gain important leadership skills relevant to their careers, such as interpreting statutes, navigating political power, engaging with their community, developing strategy, presenting to the media, and others. Physicians and other health professionals are rarely formally trained in these subjects although building health-related organizations requires strong leadership abilities. Further, students can have significant public influence and gain access to policymakers often denied to other segments of the public because they are entering respected professions. Although this differential access to power is unfair, it exists, and students can use what privilege they have to benefit others who must suffer with little recourse to changing policy. Thus, students have some advantages in advocating for harm reduction, and students may both advance an important public health agenda while developing skills that are otherwise rarely taught during training. Our experience in Orange County was that our position as health professions students offered us the legitimacy needed to build a supportive coalition, raise funds, and do the other work necessary to convince public health authorities to approve our NEP despite opposition from police and county officials.

## Working with their community

All organizers, and especially students, must build trust with PWID and the surrounding harm reduction community. This requires learning from and following their guidance, forming relationships of equality distinct from the traditional, hierarchical physician-patient and mentor-protégé relationships experienced by most students. Harm reduction programs were first invented and promoted by PWID, not professionals, and NEPs and other harm reduction programs should involve PWID in their leadership and operations. Organizers, especially those who have not used injection drugs, will need to develop relationships where they learn from PWID about their unique circumstances and needs, and work together using the resources they each have to develop a successful program.

Collaborative relationships where differences are recognized, appreciated, and used for the betterment of all parties are typically enjoyable, encourage participation, and allow a range of views to be discussed and compared. In contrast, relationships with sharp differences in power often enforce conformity and sycophancy, leaving the subordinate silenced, afraid to voice concerns, and with disturbing frequency, bullied or mistreated. Public health as a field has begun to see the benefits of a collaborative, non-hierarchical (or at least less hierarchical) approach [23], but medicine and other professions have yet to catch up. When students gain direct experience with more egalitarian ways of interacting with future patients and colleagues that they would not have experienced in standard clinical environments, they are more likely to be capable of improving the culture of their profession throughout their career.

Further, people who are motivated to become NEP staff are typically diverse, including former PWID, their families, other students, and a broad range of individuals from the surrounding community. Student organizers themselves can come from many different fields, with students thus naturally gaining experience working with inter-professional teams, and with program development benefiting from different perspectives, skills, and knowledge. As an example, our program includes students in medicine, criminology, law, public health, public policy, nursing, biology, and many other fields, with levels of study ranging from undergraduate through dual-doctoral degree programs. To use the jargon of our time, this exemplifies the patient-centered, multidisciplinary team approach now often celebrated but infrequently practiced in medicine.

## Overcoming stigma

Stigma against PWID, homeless people, sex workers, transgender people, undocumented immigrants, and other marginalized groups that benefit from NEPs exist in medicine, and negative attitudes of current practitioners are seen and internalized by students. Addiction and drug-related policy issues are not well-addressed in most students’ training, leaving students’ beliefs to be predominantly influenced by other factors, such as politics [21]. A powerful method to overcome internalized stigma and lack of training is for students to have positive personal experiences with people from those groups which then challenge the biases they encounter from their mentors. Frequently we hear volunteers at our NEP state their surprise at the positive, kind, friendly, and often fun environment found there and in many other harm reduction organizations. Over time, students talk to NEP clients and directly learn about their experiences of direct and structural violence, trauma, and the origins of their substance use (or reasons for gender transition, or lack of access to insulin syringes for undocumented clients, to name two reasons unrelated to drug use that cause clients to use our NEP). As NEPs focus on empowerment, rather than treatment, students participate in a nonstigmatizing relationship which they can bring back to their workplaces. Empathetic and nonjudgmental education, encouraging any positive change, and discussing topics often tabooed in society are essential aspects of harm reduction work, and students over time become grounded and comfortable in these practices. Depending on the services offered, students may become proficient in wound care and abscess drainage, concrete clinical skills useful in many health fields. Often, we see students leave with an increased desire to work with substance-using patients and a deeper understanding and patience for the struggles they experience. We are not aware of other environments which generate these sentiments with similar frequency and impact.

## Conclusion

Professional and graduate students have the potential to become organizers and leaders of harm reduction organizations. Our experience is that NEPs are excellent public health programs for students to build, operate, and advocate for. NEPs provide many educational benefits to students, including practical political skills, deeper understanding of structural inequalities, improved communication and education skills, appreciation of alternative modes of interacting with patients and colleagues, and logistical ingenuity. Student-organized NEPs benefit the community in a clear way: by reducing the risk of contracting infectious diseases and improving linkage to care among PWID. As NEPs are not logistically complicated interventions, they are able to have large impact without significant cost, medical licenses, or the infrastructure required of medical clinics. The primary challenges students may encounter are a lack of familiarity with harm reduction and political opposition, the first of which can be overcome by engagement with other harm reductionists and PWID, and the second of which can often be circumvented, overturned, or avoided by building harm reduction programs which do not include banned elements while continuing to advocate for change. We encourage students and their mentors to consider learning about harm reduction and organizing an NEP as an important way to benefit their community and develop as health professionals.

## Abbreviations

NEP: Needle exchange program
PWID: People who inject drugs
